# Supplementary material for: Collaborative intelligence in AI: Evaluating the performance of a council of AIs on the USMLE
Source: PLOS Digit Health. 2025 Oct 9;4(10):e0000787. doi: 10.1371/journal.pdig.0000787 (PMC12510544; doi:10.1371/journal.pdig.0000787)
Supplement: S2 File — (ZIP) [file pdig.0000787.s002.zip › S2 Data Raw data files containing unprocessed question inputs and LLM outputs including transcripts of deliberations/STEP3_Question-65-1_Type-MC-NJ.docx]

**Facilitator** **[Round 1]:** A randomized controlled trial is conducted to assess the effectiveness of a new combination-drug antihypertensive therapy (Drug X) compared with a standard antihypertensive single-drug therapy. Study participants include 140 women (70%) and 60 men (30%) ages 30 to 60 years, with baseline blood pressure measurements of 150/95 mm Hg or higher. The investigators defined antihypertensive therapy as effective if the treatment resulted in a blood pressure measurement below 140/90 mm Hg. When designing the study, the investigators set the probability of wrongly finding that Drug X is more effective than the standard therapy as 1%; they set the probability of wrongly finding that the effectiveness of the two drugs is the same as 10%. Which of the following is the most accurate estimate of the statistical power in this study? (A) 1% (B) 10% (C) 40% (D) 90% (E) 99%

**AI_C:** (1) RESTATING THE QUESTION: You're seeking to understand the statistical power of a controlled clinical trial that was designed to evaluate the efficiency of a new combination antihypertensive therapy (Drug X), as compared to a standard, single-drug antihypertensive therapy. Essentially, the study aims to determine whether Drug X is more effective at reducing blood pressure than the standard treatment. Efficiency is defined as achieving a blood pressure level below 140/90 mm Hg. The investigators set a 1% likelihood of incorrectly concluding that Drug X is superior and a 10% likelihood of falsely asserting that the two treatments have the same effectiveness. (2) PLANNING: We must ascertain the statistical power of the study. In statistics, power is generally defined as the probability that a test correctly rejects a false null hypothesis (i.e., the probability of not committing a Type II error). In this case, Type II error refers to the situation where the two drugs actually have different efficacies, but the study concludes they are equal. From the question, it is stated that the probability of a Type II error (also known as Beta error) is 10%. (3) CLINICAL REASONING: We need to calculate the power (1 - β) of the study, where β is the probability of making a Type II error. In this case, where β (type II error) is 10%, we must subtract this from 100% to get the statistical power of the study. (4) CONCLUDING: After performing these calculations, we find that the power of the study is 90%. (5) RESPONSE: Therefore, according to our analysis, the most accurate estimate of the statistical power in this study is 90% (D).

**AI_A:** (1) RESTATING THE QUESTION: The scenario describes a randomized controlled trial that was conducted to assess and compare the effectiveness of a new antihypertensive medication, Drug X, to a standard single-drug treatment. The study's sample comprises both men and women age 30 to 60 years with a baseline blood pressure reading of 150/95 mm Hg or higher. The effectiveness of a treatment is defined by the results in lowering blood pressure to a level below 140/90 mm Hg. The investigators set two error thresholds: 1% for falsely concluding that Drug X is more effective, and 10% for falsely concluding that both drugs have the same efficacy. The question is asking for the most accurate estimate of the study's statistical power. (2) PLANNING: In order to answer this we need to understand a few definitions. Statistical power is the probability that a study will reject the null hypothesis when it is false, i.e. detect a difference when one actually exists. This is known as Type II error (failing to reject the null hypothesis when it is false). The threshold set for this type of error in this study is 10%. Therefore, Statistical Power = 1 - the probability of a Type II error. (3) CLINICAL REASONING: In this study, the probability of committing a Type II error (also known as β) was predetermined as 10%. This would occur if they falsely concluded that the new and standard antihypertensive drugs had the same effectiveness when, in fact, Drug X was more effective. Given this, we can use the equation for statistical power, which is 1 - β. (4) CONCLUDING: Since β (Type II error) is set at 10%, we subtract this from 100% to arrive at the study's statistical power. (5) RESPONSE: Based on the planned assumption and the clinical reasoning used, the most accurate estimate of the statistical power of this study is 90% (D).

**AI_E:** RESTATING THE QUESTION: This question is essentially asking about the approximate statistical power of the trial study, given the set probabilities of type I and II errors. Type I error, also referred to as "false positive," is when we incorrectly reject a true null hypothesis--in this context, this means mistakingly concluding Drug X is more efficient than the standard therapy. The researchers have set this probability at 1%. Type II error, or "false negative," would be incorrectly accepting a false null hypothesis--meaning here, incorrectly concluding that the two drugs are equally effective. This probability has been set at 10% in this case. PLANNING: First, it's important to understand that statistical power refers to the probability that a test will reject a false null hypothesis or the likelihood of the study correctly finding a true effect (if it exists). Consequently, the power of a statistical test is calculated as 1 minus the probability of making a Type II error (β). In this trial, the probability of β was predetermined at 10%. Now, when we question the pre-set assumptions, the main alternative hypothesis is that the statistical power of the study could be different from the pre-set chances. CLINICAL REASONING: In this question, we need to apply our understanding of the definitions and concepts mentioned in the planning stage to solve the problem. The statistical power of a study is simply the ability of the research design to correctly identify real effects and reject the null hypothesis when it's false. In our case, the study would fail to identify the efficacy of Drug X over the standard drug if it commits a type II error. CONCLUDING: From understanding these relations, we can use the formula to predict the statistical power, which computed as (1-β), and since β was pre-set at 10% (or 0.10 in decimal form), we would calculate power as (1 - 0.10) or equivalently, (1 - 10%) in percentage terms. RESPONSE: Therefore, in this context, the statistical power of this study should be about 90%. Hence, the correct answer should be (D) 90%.

**AI_B:** RESTATING THE QUESTION: This question inquires about a randomized controlled trial comparing the effectiveness of a new combination-drug antihypertensive therapy (Drug X) and a standard antihypertensive single-drug therapy. The trial involves variables such as participant gender and age, baseline blood pressure, and treatment outcomes. The investigators defined the probability of two types of errors: falsely concluding that Drug X is superior (1%) and falsely concluding that both drugs are equally effective (10%). The question asks for the approximate statistical power, which refers to the likelihood that a study will detect a true effect when one actually exists. PLANNING: The assumption here is that statistical power is determined by the desired level of confidence to avoid type II errors (i.e., failing to detect a true effect). Alternate assumptions could involve statistical power being related to the proportion of participants, their baseline data, or the effectiveness of the therapies. However, statistically, power is calculated as 1 minus the probability of making a type II error, also known as the beta error (β), making this the preferred assumption for this question. CLINICAL REASONING: From a clinical investigation standpoint, it's crucial to understand the significance of power and error types. A Type I error (alpha/α) occurs when a true null hypothesis is rejected (false positive), signaling that Drug X is more effective when it isn't. A Type II error (beta/β) arises when a false null hypothesis is not rejected (false negative), indicating that the effectiveness of both drugs is the same when Drug X is actually superior. In this case, the probability of type II error (β) is set at 10%, and because statistical power equals 1 - β, we can calculate the power. CONCLUDING: After analyzing the relationship between the power and probability of the type II error, we ascertain that statistical power is calculated based on the predetermined probability of a type II error. RESPONSE: Thus, the most accurate estimate of the statistical power in this study would be 90% (D), which can be derived by subtracting the probability of a Type II error (10%) from 100%.

**AI_D:** 1) RESTATING THE QUESTION: The request is asking for the most accurate estimate of the statistical power of the study described. The study is a randomized controlled trial comparing the effectiveness of a new combination-drug antihypertensive therapy (Drug X) to a standard antihypertensive single-drug therapy. The investigators set up the study so that there was a 1% risk of falsely concluding that Drug X is more effective than the standard therapy, and a 10% risk of falsely concluding that both drugs are equally effective. The "statistical power" refers to the probability that the test correctly rejects the null hypothesis when the alternative hypothesis is true. In simpler terms, the statistical power is the likelihood that, if Drug X truly is more effective than the standard therapy, the study will correctly find this. 2) PLANNING: We need to take into account that the risk of Type I error (alpha), defined in this study as falsely concluding that Drug X is more effective, is set at 1%, and the risk of Type II error (beta), defined in this study as falsely concluding that both drugs are equally effective, is set at 10%. Our first assumption is that the statistical power of a study is correlated with the risks of type I and type II errors. Assuming that alpha and beta are directly related, we can hypothesize that the statistical power (1-beta) would be at the opposite percentage of beta (Type II error). 3) CLINICAL REASONING: In statistical terms, a Type I error (alpha) occurs when we reject a true null hypothesis – meaning, in this case, we would be falsely declaring Drug X superior to the standard therapy. Conversely, a Type II error (beta) happens when we fail to reject a false null hypothesis – or, in this study, we wrongly conclude that both drugs are equally effective. The power of the study is calculated as 1 - beta and represents the likelihood that the study will detect a difference between the two therapies if one truly exists. 4) CONCLUDING: Since the investigators set the chance of making a Type II error (falsely assuming both drugs to be equally effective) at 10%, the statistical power of the study, which is the probability of correctly identifying Drug X as more effective when it truly is (1-beta), would be 90%. 5) RESPONSE: Therefore, the most accurate estimate of the statistical power in this study is (D) 90%.

**Facilitator** The question describes a randomized controlled trial that aims to compare the effectiveness of a new antihypertensive medication, Drug X, to a standard single-drug treatment. The effectiveness of a treatment is defined by its ability to lower blood pressure to a level below 140/90 mm Hg. The investigators have set two error thresholds: 1% for falsely concluding that Drug X is more effective, and 10% for falsely concluding that both drugs have the same efficacy. Statistical power is the probability that a study will reject the null hypothesis when it is false, i.e., detect a difference when one actually exists. This is known as avoiding a Type II error (failing to reject the null hypothesis when it is false). In this study, the probability of committing a Type II error (also known as β) was predetermined as 10%. This would occur if they falsely concluded that the new and standard antihypertensive drugs had the same effectiveness when, in fact, Drug X was more effective. Given this, we can use the equation for statistical power, which is 1 - β. From a clinical investigation standpoint, it's crucial to understand the significance of power and error types. A Type I error (alpha/α) occurs when a true null hypothesis is rejected (false positive), signaling that Drug X is more effective when it isn't. A Type II error (beta/β) arises when a false null hypothesis is not rejected (false negative), indicating that the effectiveness of both drugs is the same when Drug X is actually superior. After analyzing the relationship between the power and probability of the type II error, we ascertain that statistical power is calculated based on the predetermined probability of a type II error. Since β (Type II error) is set at 10%, we subtract this from 100% to arrive at the study's statistical power. Therefore, based on the planned assumption and the clinical reasoning used, the most accurate estimate of the statistical power of this study is 90% (D). This can be derived by subtracting the probability of a Type II error (10%) from 100%.
